# The Ebola Crisis and the Corresponding Public Behavior: A System Dynamics Approach

**DOI:** 10.1371/currents.outbreaks.23badd9821870a002fa86bef6893c01d

**Published:** 2016-11-03

**Authors:** Nasser Sharareh, Nasim S. Sabounchi, Hiroki Sayama, Roderick MacDonald

**Affiliations:** Systems Science and Industrial Engineering, Binghamton University, Binghamton, New York, USA; Systems Science and Industrial Engineering, Binghamton University, Binghamton, New York, USA; Systems Science and Industrial Engineering, Binghamton University, Binghamton, New York, USA; Rockefeller College of Public Affairs and Policy, University at Albany, State University of New York, Albany, New York, USA

## Abstract

Background: The interaction of several sociocultural and environmental factors during an epidemic crisis leads to behavioral responses that consequently make the crisis control a complex problem.

Methods: The system dynamics approach has been adopted to study the relationships between spread of disease, public attention, situational awareness, and community’s response to the Ebola epidemic.

Results: In developing different simulation models to capture the trend of death and incidence data from the World Health Organization for the Ebola outbreak, the final model has the best fit to the historical trends. Results demonstrate that the increase of quarantining rate over time due to increase in situational awareness and performing safe burials had a significant impact on the control of epidemic. However, public attention did not play a significant role.

Conclusion: The best fit to historical data are achieved when behavioral factors specific to West Africa like studying the Situational Awareness and Public Attention are included in the model. However, by ignoring the sociocultural factors, the model is not able to represent the reality; therefore, in the case of any epidemics, it is necessary that all the parties and community members find the most significant behavioral factors that can curb the epidemic.

## Introduction

The 2014 Ebola outbreak was the deadliest in history affecting three countries in West Africa. The virus spreads through direct contact with body fluids of infected individuals, or objects that have been contaminated by an infected person. The Ebola virus is considered a behavioral disease by USAID (2015)^1^ and WHO (2015b)^2^ as the transmission mechanism is greatly influenced by the behavior of individuals, families, and communities. As a result disease transmission not only occurs in healthcare facilities, but also among family members while the person is sick and during funeral practices after death. Since traditional funeral rituals, in West Africa, require washing, touching and kissing the body of the deceased, the virus spreads rapidly after death, just when family members have the most contact with the body. Thus, the contribution of sociocultural factors in the Ebola epidemic is significant. Our objective is to study the interaction of the sociocultural and environmental factors during the recent Ebola epidemic crisis for the purpose of designing more effective control strategies of the epidemic.

Some studies show the effect of different behavioral factors and their positive or negative impacts on the spread of Ebola. According to Pruyt et al. (2015)^3^, some psychological and sociocultural factors have adverse effects on the spread of Ebola while some can balance the outbreak, such as increasing hygiene, or reducing the contact rate. Pruyt et al. (2015)^3^, developed a simulation model to test various control strategies and concluded that due to the adverse effects of sociocultural factors, the number of cases may rise up to 4.6 million, but if we redirect some psychological and sociocultural behavior, it will curb the outbreak by the end of December 2014, which shows the importance of studying the sociocultural factors in controlling the epidemic.

Several studies discuss strategies and interventions to fight Ebola. According to Gimm and Nichols (2015)^4^, two main strategies to stop the spread of Ebola, are to diagnose people who are infected or in danger of getting infected, prevent further disease spread by quarantining the symptomatic individuals, trace the contacts of infected ones, and hold safe burials for the dead. Another strategy is to develop effective vaccines and treatments. In contrast, Calain and Poncin (2015)^5^ mention that isolation and quarantining might violate the human rights and may have counterproductive effects on public’s behavior.

Furthermore, there are many difficulties during the process of quarantining people in Africa according to Kutalek et al. (2015)^6^. Reasons of resistance by families of those quarantined include the absence of food for quarantined people that forced them to break the quarantine to purchase food, absence of good communication methods that preventing people from knowing the condition of those quarantined, and the absence of appropriate support for families who lost one of their members. Thus, in order to facilitate the process of quarantining and obtaining better results, one needs to have a better understanding of the cultural values and design more effective interventions by understanding the underlying assumptions.

Simulation models are extremely helpful for testing different policy scenarios during such crises (Merler et al., 2015)^7^ by saving time in disease management and in mitigating the spread of disease. Also, the lack of proper simulation models can be quite costly when policies are implemented and unforeseen consequences arise (Thompson et al., 2015)^8^. For example, although, media can play a positive role during mass emergencies by collecting and disseminating useful information gathered from the public (Latonero and Shklovski, 2011)^9^, in some cases, running public awareness ads and the release of information by news agencies without considering the knowledge level of the public or through the wrong media (TV as opposed to social media), may create unnecessary fear and misinformation (Smith, 2006)^10^. On the other hand, as discussed by Rowell (2014)^11^, in the case of the spread of a fatal disease, such as Ebola, it is not wise to eliminate public fear; on the contrary, the author explains that fear can be leveraged to increase public awareness about the proper steps to reduce transmission of the disease. Government officials and policy makers should have considered this notion during the recent Ebola outbreak (Rowell, 2014)^11^.

A simulation model that fits historical data trends on the number of deaths and incidence, while capturing the social and behavioral factors, can help with analyzing various policy scenarios prior to implementation, and decrease costs of unintended consequences. Overall, considering that the Ebola crisis is a complex problem due to the interaction of several sociocultural, and environmental factors over time, a system dynamics (SD) approach provides a promising framework for studying this problem. SD simulation models have also been applied to epidemics and other complex public health issues including eradication of polio (Thompson et al., 2015)^8^, and dealing with obesity (Rahmandad and Sabounchi, 2012)^12^.

In the following section, some background information for the modeling process is discussed and the key historical trends of the spread of Ebola are presented. Later, the causal loop diagram is provided that shows the important causal relationships between different key factors, which had a significant influence on public’s situational awareness and attention towards the epidemic and consequently the spread of Ebola. Also, the corresponding causal structure that represents the trends observed from tweet data is discussed to understand the relationship between the spread of Ebola in West Africa and behavioral response to disease among the population. Then in the simulation section, the final model, calibration results and fitness measures with historical trends is thoroughly described. Finally, the conclusion with a summary of the main insights of our research and future research directions will be discussed.

## Methodology and Model Development

In this paper, a system dynamics (SD) approach is used to study the impact of social and behavioral factors on the spread of Ebola, and also to identify how people’s perceptions about the situation can have different effects on controlling the outbreak. System dynamics approach focuses on understanding the relationship between the structure of a system and the resulting dynamic behaviors generated through multiple interacting feedback loops (Sterman, 2000)^13^. This approach is especially useful in explaining why systems, in general, are resistant to policy implementation and planned change, and produce mathematical models that allow policy makers to understand the potential effects of alternative strategies and use the information to make better decisions regarding policy and program directions.

In order to develop the system dynamics model, we identify the main causal forces that lead to the epidemic and illustrate within the Causal Loop Diagram (CLD) (Figure 2). The CLD demonstrates the major feedback loops that we hypothesize are responsible for the growth and decline of the number of infected and death from Ebola. However, a CLD is not a simulation model. Any system dynamics simulation model can be expressed as a system of differential equations. When using software such as vensim (Ventana System, 2014)^14^ to simulate the model, the differential equations are hidden from the user and we only need to define the stocks and flows instead of state variables and state derivatives. In other words, we develop Stock and Flow Diagram (SFD) to run simulations and test the validity of our initial hypothesis defined in the CLD.

Stock variables represent accumulations and define the state of the system (Rectangles in Figure 3). We define stock variables by integrating its net flow rate that is the difference between the inflow and the outflow rates of a process, over a period of time (Sterman, 2000)^13^.We use equations from the Susceptible-Infected-Recovered (SIR) model (Kermack and McKendrick, 1927)^15^ to define the mathematical equations for contagion and depletion flow rates for the Ebola epidemic. Also, we adopt a third and fourth order information delay structure to capture the changes in public attention and situational awareness based on the trends in tweets posted.

The simulation results of the model should replicate the dynamics of the 2014 West African Ebola outbreak. We used the WHO situation reports (WHO, 2015a)^16^ to find the number of incidence of Ebola and the total cumulative number of deaths that occurred in Guinea, Liberia, and Sierra-Leone from March 2014 to March 2015. Also, the social media site Twitter is used as a source for behavioral and societal feedback monitoring. Various modeling studies have used tweet data to capture social and behavioral dynamics during epidemics (Odlum and Yoon, 2015)^17^. Figure 1 shows the number of Ebola deaths (the circles) based on WHO data sources along with the number of tweets (the triangles) made each month for different hashtags relevant to Ebola including [#Ebolafacts, #Ebolaoutbreak, #Ebolavirus, #FightingEbola, and #StopEbola] based on data collected by Symplur, the healthcare social media analytics company (Symplur, 2015)^18^. In our model, we use the tweets trend as an initial estimate of the spread of attention among the public. The x-axis is the number of months, from March 2014 (i.e. Month 1) to Feb 2015 (i.e. Month 12).

**Number of death of Ebola vs number of tweets – x-axis: months from March 2014 to March 2015 – right y-axis: number of deaths Source: (WHO 2015), left y-axis: number of tweets Source: Symplur 2015:**
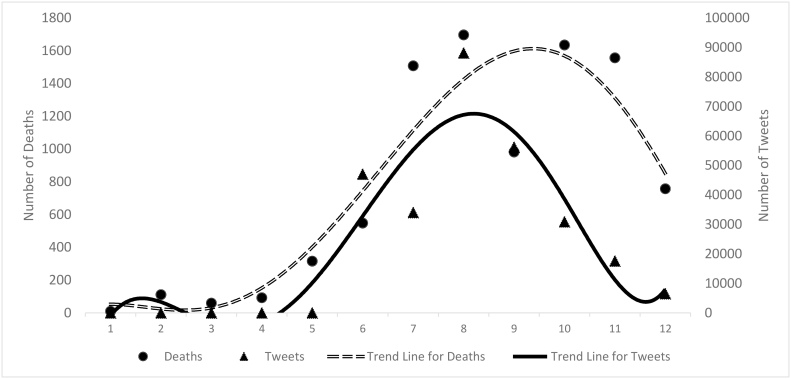

In Figure 1, a trend line has been fitted to the number of tweets for Ebola. The trend shows that tweets increased rapidly while Ebola was spreading, but then tweets slowed down after a 4-months period. In November 2014 tweets started dropping from 88,000 to 6,500 tweets in Feb 2015. This drop in number reflects that some underlying reinforcing mechanism, led to exponential growth of tweets on Ebola, and then later some balancing mechanism slowed that growth. After a while, the tweets, or concerns and public attention has dropped dramatically.

***Causal Loop Diagram***

In the first phase of our modeling process, a qualitative model called a Causal Loop Diagram (CLD) (Sterman, 2000)^13^ was developed by reviewing relevant literature to understand the causal mechanism underlying the changes in attention and awareness and the subsequent impact on the spread of Ebola (See Figure 2). The diagram outlines the feedback structure and our dynamic hypothesis in describing the data trends presented in the previous section. In this diagram, a plus sign on an arrow means that change in the cause variable leads to a change in the same direction for the effect variable. Also, a negative sign means that the effect variable changes in the opposite direction of the cause variable. Once the links form a loop, if the number of negative signs in a loop is even, then that loop is a reinforcing loop which leads to an exponential growth or decline, otherwise, it is a balancing loop which leads to an increasing or decreasing goal seeking behavior. For instance, the reinforcing feedback loop denoted by R1 in Figure 2, which is known as the contagion loop in the Susceptible-Infected-Recovered (SIR) model (Sterman, 2000)^13^, shows that as the number of infected by Ebola grows, the infection rate increases and consequently generates an exponential growth in the number of infected. In addition, as the number of infected increases, death toll and the number of unsafe funerals will rise and consequently will increase the infection rate (Loop R2). Also, in the balancing feedback loop B1, which is known as the depletion loop in the SIR model, as the susceptible population decreases, infection rate will decline and the number of infected will gradually level off.

Furthermore, the impact of Situational Awareness on reducing the infection rate can be visualized in feedback loops B2 and B3. ‘Situational Awareness (SA)’ was originally defined to explain the jet pilot’s need to have a continuous awareness about his environment during flight (Stanton, Chambers, and Piggott, 2001)^19^. In simple terms ‘Situational Awareness (SA)’ is defined as proper awareness of a situation through dynamics interaction between human and their environment, that consists of three levels including perception of environmental elements in both time and space dimensions, reflection of the situation in a conscious dynamic manner and prediction of future status (Stanton, Chambers, and Piggott (2001)^19^. Situational Awareness generates the required knowledge and behavior in interaction with the environment to achieve the specified goals. It can be enhanced by information and critical cues on the current status of the situation, if presented in a way that makes different levels of SA including understanding and prediction easier (Stanton, Chambers, and Piggott (2001)^19^. Also, a poor degree of Situational Awareness (SA) has a counterproductive effect on people’s ability to detect problems and act accordingly Stanton, Chambers, and Piggott (2001)^19^.

During the Ebola epidemic, the Situational Awareness (SA) of people increases when the number of deaths reported increases. Also, training people about the disease and prevention approaches by community members and WHO (Abramowitz et al., 2015)^20^, will help people to act properly at the time of emergency and increase their situational awareness (Loops B5 and B6). As Situational Awareness (SA) increases, there will be less resistance towards quarantining and hospitalizing symptomatic individuals and more people will be quarantined and hospitalized in a timely manner. Also, there will be fewer unsafe funerals performed, and consequently after a while, the infection rate will drop (Loop B2).

In addition, as the number of deaths reported increases, fear among the population and consequently public attention towards Ebola increases. As people’s attention grows, they will avoid going out in public, which will decrease the contact rate and consequently reduce the spread of the disease (Loop B4). Furthermore, during the epidemic, a massive amount of news was broadcasted regarding Ebola and everyone was talking about Ebola, its symptoms, and strategies to avoid getting infected. Some media coverage was releasing news with misinformation on Ebola or made the spread of disease sound too dramatic without providing clear information on prevention and dealing with the disease; like the possibility of transmission of disease through the air (Rahmandad and Sabounchi, 2012)^12^. This whole process reinforced fear among the population, which is captured by feedback loop R5 in Figure 2. More interestingly, public attention towards the disease can be reinforced by the media coverage because people will demand more news about the disease (Loop R4). As evidence, during these controversial headlines in the news, twitter experienced a huge number of tweets about fear and Ebola (Odlum and Yoon, 2015)^17^. Also, Odlum and Yoon (2015) mention that after the first announcement from the CDC about the Ebola outbreak in Africa on July 28th, 2014, the number of tweets increased sharply.

**Causal Loop Diagram:**
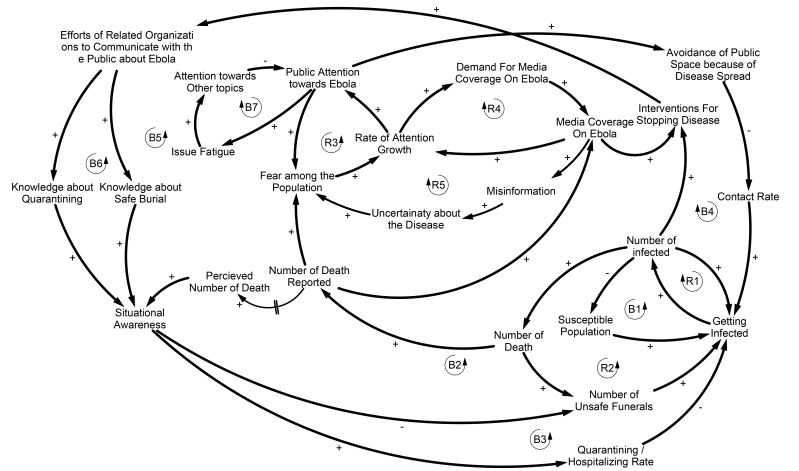

In summary, based upon the diagram in Figure 2, our hypothesis is that during the first months of the outbreak, public attention towards Ebola was spreading due to uncertainty in news reporting, fear among the population, and due to an incorrect perception of people about Ebola. However, the public attention to new issues, such as the Ebola outbreak, has a trend that increases exponentially followed by a sudden fall; this pattern is common and is referred to as issue fatigue (Waldherr, 2014)^21^ that is captured in Loop B7 of Figure 2. This pattern is also observed in the number of tweets that peaked and declined while the number of deaths was still increasing (See Figure 1). In order to simplify reading the diagram, the components of each loop are mentioned in Table 1.

**Table 1:** Loop's elements

Loop Name	Components
R1	Getting infected => Number of infected
R2	Getting infected => Number of infected => Number of death => Number of unsafe funerals
R3	Public attention towards Ebola => Fear among the population => Rate of attention growth
R4	Rate of attention growth => Demand for media coverage on Ebola => Media Coverage on Ebola
R5	Rate of attention growth => Demand for media coverage on Ebola => Media Coverage on Ebola => Misinformation => Uncertainty about Disease => Fear among the population
B1	Getting infected => Number of infected => Susceptible population
B2	Situational Awareness => Number of unsafe funerals => Getting infected => Number of infected => Number of death => Number of death reported => Perceived number of death
B3	Situational Awareness => Quarantining / Hospitalizing Rate => Getting infected => Number of infected => Number of death => Number of death reported => Perceived number of death
B4	Getting infected => Number of infected => Number of death => Number of death reported => Fear among the population => Rate of attention growth => Public attention towards Ebola => Avoidance of public space because of disease spread => Contact rate
B5	Situational Awareness => Number of unsafe funerals (Quarantining / Hospitalizing Rate) => Getting infected => Number of infected => Interventions for stopping disease => Efforts of related organizations to communicate with the public about Ebola => Knowledge about Safe Burial
B6	Situational Awareness => Number of unsafe funerals (Quarantining / Hospitalizing Rate) => Getting infected => Number of infected => Interventions for stopping disease => Efforts of related organizations to communicate with the public about Ebola => Knowledge about Quarantining
B7	Public attention towards Ebola => Issue Fatigue => Attention towards other topics

## The Simulation Model

In our modeling approach, system behavior is described based on the underlying causal relationships and feedback structure. In order to test our dynamic hypothesis outlined in the causal loop diagram in explaining the dynamic behavior trends of Ebola deaths and cases, a simulation model is developed. The model is an extended variation of the Susceptible-Infected-Recovered (SIR) model (Kermack and McKendrick, 1927)^15^ that is expanded to capture psychological and social behaviors identified in this epidemic. Although no model is 100% valid; there are many ways to test the validity of a model including extreme condition tests, boundary adequacy tests, and sensitivity analysis. The main goal of these tests are to see if the right dynamic problem has been identified, if the right behavior for the right reasons has been modeled, and if the model is useful in a specific context (Sterman, 2000)^13^ the final validated model is calibrated based on time series data on number of death and incidence collected by WHO (WHO, 2015a)^16^. This model is an attempt to include social and behavioral factors in studying the epidemic, which can be used as a framework for further policy analysis.

Six different simulation models were developed, starting from the basic SIR model (Sterman, 2000)^13^. The objective is to replicate the historical number of deaths and cases from WHO data. As we further revise the model and add more details of underlying social and behavioral mechanisms to the model, the simulation results better fit the historical trends and also the model explains more precisely the events during the epidemic. The purpose is to understand, which model and additional structure has the most significant influence on the output of the model and makes it fit better with historical trends. After SIR model, the next model consists of those three stocks in SIR mode- susceptible, infected, and recovered- plus death stock. Now we needed to know what the average of the death of people was during the outbreak. In the next model, we added the quarantined stock to the model, therefore, we needed to know the percentage of people who get quarantined. In the next version, we considered this percentage as a dynamic parameter which can be increased during the time, since people were showing less contrast in getting quarantined as they were getting more knowledge about the disease. In the fifth version, we added asymptomatic population stock to the model to capture the incubation time. So far, different kinds of population stocks have been captured and some social and behavioral factors like situational Awareness (which will be explained in detail later) have been added to the model, but in the final version, the effect of public attention on contact rate has been added too which will be explained later. In table 2, you are provided with all these steps. It is also vital to know that, although, modeling is the art of simplification, but we tried to add as many details as possible to the model to consider the most important factors and get closer to the reality.

**Table 2:** Models development process

Model	Stocks	Descriptions
1 (SIR Model)	Susceptible – Infected - Recovered	Simple SIR model with constant contact rate over the time
2	Added Stock: Death	Capturing the death rate and considering the reduction of contact rate over the time
3	Added Stock: Quarantined	Investigating the influence of quarantining the infected on reducing the death rate
4	Added Stock: Perception of Death	Capturing the increase of situational awareness which leads to more safe burials
5	Added Stocks: Asymptomatic and Symptomatic	Capturing the incubation time to separate the symptomatic individuals from asymptomatics, and more precisely define the infection rate
6 (Final Model)	Added Stock: Public Attention	Studying the influence of public attention on contact rate by incorporating twitter data trends

The final model consists of seven population stocks including Susceptible, Infected that are Asymptomatic and Symptomatic, Quarantined and Hospitalized, Recovered and the Dead that are either buried or yet in the stage of getting buried (See Figure 3).

In this model, the effect of tweets or public attention on contact rate has been added to the model. Public attention increases according to public information about the death toll (See Figure 3). However, as death rate declines, public attention will also drop; consequently, its corresponding effect on tweets has the same behavior. Also, Public Attention influences the contact rate between the infected and susceptible population. As public attention increases, contact rates decline. But, after a while people start to have their routine life and contact rate increases to its original value following the trend of public attention.

**Snapshot of final model:**
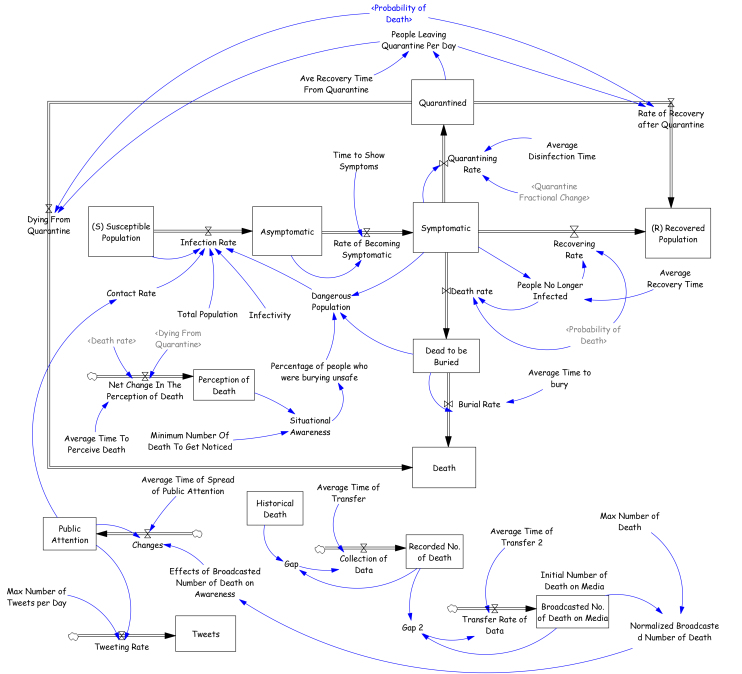

In the model, Situational Awareness (SA) is defined based on the ratio of death toll to a baseline value of ‘Minimum Number of Death to Get Noticed’. Situational Awareness (SA) modifies the traditional and unsafe funerals. When people’s SA increased, and they learned how to perform safe burials, their behavior changed and they started to avoid having unsafe rituals. Furthermore, higher situational awareness increases the willingness to get quarantined/hospitalized that is represented by the parameter ‘quarantine fractional change’. The simulated results show that there is a delay of 190 days from the beginning of the outbreak before Situational Awareness (SA) starts to increase. This concept can be explained by the concept of Normalcy Bias that makes people ignore or underestimate the disaster’s effects and it can happen for regular people and even policy makers (Moore, 2014)^22^.

***Calibration***

In order to define the parameters in the simulation model, various data sources are used including literature or reports published for the Ebola outbreaks. On the other hand, for some model parameters that are critical in determining model behavior only a reasonable range of values are reported in the literature, or no data sources are found. These parameters are determined by calibration (Table 3).

**Table 3:** Sources for Defining Model Parameters

Determined based on Available Data	Value	Determined by Calibration	Value
Average Recovery Time	7 Days	Infectivity	0.0702
Incubation Time	12.62 Days	Initial Susceptible	10,000,000 People
Initial % of people who were burying unsafe	76%	Average Time to Perceive Death	90 Days
Average Recovery Time From Quarantine	7 Days	Minimum Number of Death to Get Noticed	113 Deaths
Average Time to Bury	8.82 Days	Contact Rate	6 Contacts/Day
Average Disinfection Time	2 Days		

Some model parameters are not precisely defined but various literature reports a reasonable range of values. For example, according to Dowell et al. (1999)^23^, the incubation period in the Ebola epidemic in Congo in 1976 was 7 days and people were dying 7 to 14 days after the onset of symptoms. Also in a study conducted by Bwaka et al. (1999)^24^ they mentioned that patients were starting to recover 2 weeks after the onset of symptoms, and the fatal cases were occurring between the 7th and 10th day.

However, due to the dispersion of Ebola in different countries, the parameters that we are interested in using in the model may vary among these countries. For instance, according to Rivers et al. (2014)^25^, parameters for the Ebola outbreak in Liberia and Sierra Leone have different values shown in Table 4. For these parameters, a reasonable interval is defined based on the literature, and the best-fit estimate is found by calibration. For instance, for the “time to show symptoms” the range is [2, 21] days, with the mean of 7-8 days. After calibration, the value was estimated to be 12.62.

**Table 4:** Differences between parameter's value in Liberia and Sierra Leone

Parameters	Liberia	Sierra Leone
Contact Rate (Community)	0.16	0.128
Contact Rate (Hospital)	0.062	0.08
Contact Rate (Funeral)	0.489	0.111
Incubation Period	12 Days	10 Days
Time from Hospitalization to Recovery	15.88 Days	15.88 Days
Time from Hospitalization to Death	10.07 Days	6.26 Days
Time until Hospitalization	3.24 Days	4.12 Days

## Experimental Results

The simulation model is calibrated against data collected on the number of deaths and Ebola cases from the WHO situational reports (WHO, 2015a)^16^. The simulation period starts in January 2014 corresponding to start of the epidemic when patient zero of the Ebola outbreak, a 2-year old boy died (WHO, 2015a)^16^. The simulation runs for a total of 512 (days), until the end of May 2015 when WHO announced Liberia free of Ebola. This model is calibrated in two phases. First we calibrate the model components including ‘public attention’ to fit the S-Shaped growth and then reverse S-Shaped decline pattern of tweets made on Ebola hashtags which has been gathered during 2014 (Figure 1) and is being used as a sample to represent the West African countries public attention to the Ebola (See bottom of Figure 3). In the second phase, the rest of the model is calibrated to fit the trends of death and incidence data. The maximum likelihood estimation approach is used for calibrating the models (Dogan, 2004)^26^. In each iteration of the calibration, the objective function is minimized, which is equal to the sum of differences between the time-series data and their respective simulated values, multiplied by a weight and then squared. The weights considered are the reciprocal of the standard deviation of the prediction error for that variable, converging to the maximum likelihood estimated values of the parameters (Kermack and McKendrick, 1927)^15^.

***Result***

The final model can reasonably well replicate the time data series available on the number of death and infected cases (See Figure 4). Since the number of dead is calculated separately comparing to the original SIR model, so the results are precise in representing numbers on total recovered or died. Also, the final model captures the social and behavioral factors that had a significant influence on curbing the outbreak including the change of social contacts during the epidemic, the process of quarantining infected people, and the asymptomatic period that people do not show symptoms which are the essential parts in order to study the Ebola crisis. Therefore, the final model fits our purpose of representing the events that occurred during the Ebola outbreak.

**Cumulative number of Death and Cases vs. Historical Data from WHO:**
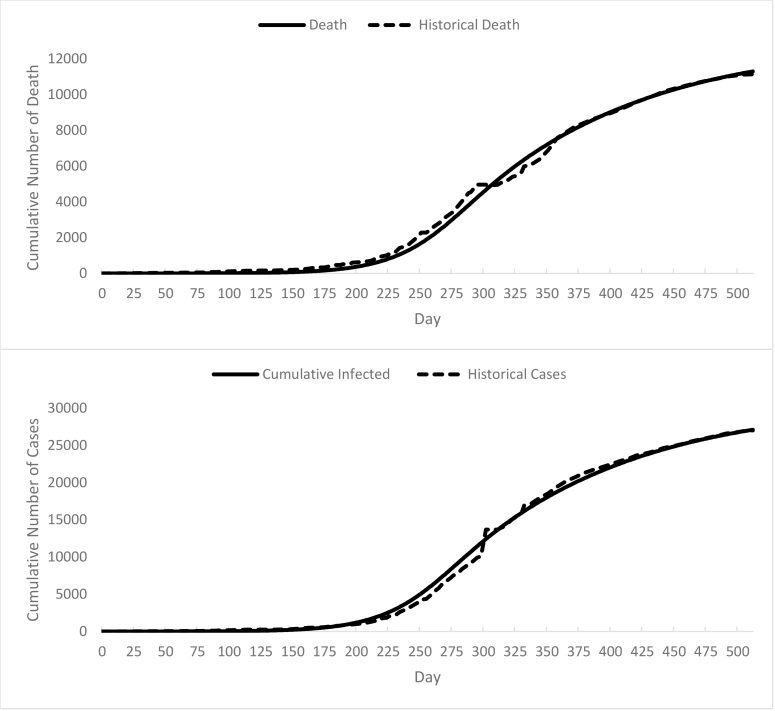

***Comparison between Models***

In order to evaluate the various versions of simulation models developed and how closely they represent the recent Ebola epidemic, the RMSE of Deaths and Cases RMSE are calculated based on the formula RMSE =

\begin{equation*}\small{}\sqrt{\sum_{1}^{n}{(x_{d} -x_{m} )^2}/n}\end{equation*}

where X_m_ indicates simulated values and X_d_ the data values.

In the final model, RMSE of deaths is 293 and is much lower than the original SIR model that has an RMSE of 1043. Also regarding the number of infected cases, final model has a RMSE of 623, while the original SIR model has 6843.

Furthermore, the final simulation model (Figure 3) has a much lower bias (0.165 for deaths and 0.06598 for infected cases) and variation (0.1282 for deaths and 0.0843 for infected cases) in comparison with other versions of models developed based on the original SIR model. For example, the original SIR model has bias value of 0.3214, and 0.4626 for deaths and infected cases respectively, and variations of 0.5589 and 0.5339 for deaths and infected cases. In conclusion, the final simulation model has the best fit in comparison with the original SIR model, which lacks behavioral and sociocultural factors, and also in comparison with the other versions of the model, final model has better fit and it is illustrating the real story in more details.

## Conclusion and Future Work

It is vital to consider how the behavior of communities plays an important role in the spread of Ebola in West Africa. This extends not only to the disease itself but also to the contagion of irrational behaviors. Not only the public, but also high ranking officials, scientists, and even policy makers are at risk for presumptive and consequently dangerous behavior; therefore, it is necessary to combine physical disease spread along with behavioral practices that hinder or spread the disease. As the United Nations head of the Ebola mission (Ismail Ould Cheikh Ahmed) attested, combatting the Ebola outbreak was slowed by both “traditional practices” and “lack of knowledge” (Ohlheiser, 2015)^27^. Future considerations of behavioral and psychological factors are needed for an adequate and effective response to outbreaks of deadly diseases such as Ebola.

The system dynamics approach is a very helpful tool in grasping the whole picture and helping key actors better understand and act upon the reality and facts within the chaotic behavioral decisions and their impact on an epidemic. Overall, response to any infectious disease such as Ebola requires constant monitoring and adaptability. Dynamic adaptability is a feature of our model, which gets away from presumptions as we strive to create a working baseline for future policy implementation modeling as well as retrospective impact assessment, particularly with regard to social and behavioral factors.

In this paper, the impact of public attention and awareness in dealing with epidemics has been studied and several system dynamics simulation models have been developed. The best model according to its ability to capture a realistic slice of reality has been provided, which is capable of generating the observed data from WHO (WHO, 2015a)^16^. In the progress of developing different variations of the simulation model so far, we have found that the sixth model has the best fit to the available time series data. This model captures the increasing trend of quarantine over time due to change in behavior that promotes quarantine and hospitalization, and suspension of traditional rituals. Also, this model more accurately captures the population that recovers or dies after leaving the infection stage.

In the future, we plan to use our best simulation model to test different policy scenarios in leveraging public fear and awareness to deal with the spread of fatal disease such as Ebola. For instance, we can study the effects of Doctors Without Borders interventions on increasing the knowledge of people and stopping the disease, to see if it truly had helped to stop Ebola outbreak in infected countries or not. Also, another direction for the future work is to further refine the model by capturing the spread of disease in the three West African countries separately and compare and contrast their disease management approaches.

In addition, system dynamics modeling approach can also be adopted to model other diseases outbreaks, but a specific model constructed for a specific disease cannot be used as is to predict other diseases, because each model contains disease-specific epidemiological, social and cultural factors. The model developed in this paper can be used in general for other contagious diseases if we are dealing with an outbreak in communities that follow similar social and cultural practices. However, we need to refine parameter values to correspond with the characteristics of the new outbreak. This requires further calibration of the model against data trends of reported infected and death numbers and also posted tweets that represent dynamics of public attention and situational awareness for the new disease outbreak.

Moreover, our current analysis of trends on tweet data on Ebola is at an aggregate level that was publicly available from a third party (Symplur, 2015)^18^. Once we obtain the actual data from twitter, we can perform content analysis and adopt data mining techniques to filter irrelevant tweets and have a more accurate analysis of the influence of sociocultural factors on people’s situational awareness and its effect on impeding the spread of Ebola.

Furthermore, by collecting field data and conducting interviews and focus group sessions with various stakeholders and affected individuals in both urban and rural communities and collecting data about their experiences from the Ebola epidemic, we will have a more precise comprehension about the sociocultural and environmental factors and further refine our model. Further, we hope to mobilize future models for the predictive potential to enhance outbreak preparedness. Such models could be used to help educate the public and key actors to create a broader awareness and collaboratively create effective protocols and disaster plans that minimize disease casualty.

## Data Availability

All the data that have been used in this study are from publicly available sources like WHO, and Symplur websites. We have referenced these sources in the manuscript. The data of Figure 1 are from Symplur, which the authors used to validate the simulation output. Total reported suspected, probable, and confirmed cases in Guinea, Liberia, and Sierra Leone have been reported by CDC, which are provided in WHO situation reports, http://www.cdc.gov/vhf/ebola/outbreaks/2014-west-africa/cumulative-cases-graphs.html. http://apps.who.int/ebola/ebola-situation-reports.

We have uploaded all these data to the Figshare repository: https://figshare.com/s/a6ac2c9cc4ec2c6dc65d (DOI: 10.6084/m9.figshare.3750774).

## Corresponding Author

Nasim Sabounchi (sabounchi@binghamton.edu) and Nasser Sharareh (nsharar1@binghamton.edu)

## Competing Interests

The authors have declared that no competing interests exist.
